# Measuring patients’ priorities using the Analytic Hierarchy Process in comparison with Best-Worst-Scaling and rating cards: methodological aspects and ranking tasks

**DOI:** 10.1186/s13561-016-0130-6

**Published:** 2016-11-14

**Authors:** Katharina Schmidt, Ana Babac, Frédéric Pauer, Kathrin Damm, J-Matthias von der Schulenburg

**Affiliations:** 1 Center for Health Economics Research Hannover (CHERH), Leibniz University of Hannover, Otto-Brenner-Str. 1, D-30159 Hanover, Germany; 2 Biomedical Research in Endstage and Obstructive Lung Disease Hannover (BREATH), Member of the German Center for Lung Research (DZL), Hanover, Germany

**Keywords:** Decision making, Analytic Hierarchy Process, Best-worst-scaling, Method comparison, Patient preferences

## Abstract

**Background:**

Identifying patient priorities and preference measurements have gained importance as patients claim a more active role in health care decision making. Due to the variety of existing methods, it is challenging to define an appropriate method for each decision problem. This study demonstrates the impact of the non-standardized Analytic Hierarchy Process (AHP) method on priorities, and compares it with Best-Worst-Scaling (BWS) and ranking card methods.

**Methods:**

We investigated AHP results for different Consistency Ratio (CR) thresholds, aggregation methods, and sensitivity analyses. We also compared criteria rankings of AHP with BWS and ranking cards results by Kendall’s tau b.

**Results:**

The sample for our decision analysis consisted of 39 patients with rare diseases and mean age of 53.82 years. The mean weights of the two groups of CR ≤ 0.1 and CR ≤ 0.2 did not differ significantly. For the aggregation by individual priority (AIP) method, the CR was higher than for aggregation by individual judgment (AIJ). In contrast, the weights of AIJ were similar compared to AIP, but some criteria’s rankings differed. Weights aggregated by geometric mean, median, and mean showed deviating results and rank reversals. Sensitivity analyses showed instable rankings. Moderate to high correlations between the rankings resulting from AHP and BWS.

**Limitations:**

Limitations were the small sample size and the heterogeneity of the patients with different rare diseases.

**Conclusion:**

In the AHP method, the number of included patients is associated with the threshold of the CR and choice of the aggregation method, whereas both directions of influence could be demonstrated. Therefore, it is important to implement standards for the AHP method. The choice of method should depend on the trade-off between the burden for participants and possibilities for analyses.

**Electronic supplementary material:**

The online version of this article (doi:10.1186/s13561-016-0130-6) contains supplementary material, which is available to authorized users.

## Background

Measurement of patient preferences and priorities has gained more relevance in health care. One reason is the increasing importance of patient participation in health care. In Germany, the Robert Koch-Institute used to call the patients “costumers” and “evaluators” in their Information System of the Federal Health Monitoring [1]. Patients also want to decide scope of service of statutory health insurances’ and which services are covered. Several studies found differences between patients’ and physicians’ perceptions of preferences (e.g., [2–5]). It is relevant to assess the preferences of the (potential) patients instead of proxy reports. Another reason for the increasing importance is the integration of preferences as utility in health economics evaluations and reimbursement decisions for pharmaceuticals. Knowledge of patients’ preferences or priorities could be a chance for optimizing the health care system according to patients’ requirements.

Decisions regarding treatment preferences must consider a variety of characteristics, so called multi-criteria decision problems. Possible options for solving decision problems are value-based methods, strategy based methods, and Conjoint Analyses (CA). The German Institute for Quality and Efficiency in Health Care (IQWiG) tested and confirmed the Analytic Hierarchy Process (AHP) method as decision making tool in health technology assessments [6]. Application of AHP for the measurement of preferences has increased during the last five years, but is still a less researched approach in health care decision making [7]. It remains unclear whether the AHP method and established decision making methods yield comparable results. Recent studies already examined the direct comparisons of AHP and CA, as seen in [8–11]. Other studies conducted comparisons between CA and Best-Worst Scaling (BWS) [12–16]. Mühlbacher and Kaczynski (2016) demonstrated the similarity of BWS results and ratings, but did not compare directly the results from AHP with BWS [17]. Although another study published by Mühlbacher et al. showed similar results for BWS and AHP methods, some of the subgroups differed in their rankings obtained by BWS and AHP method [18]. However, we found no further evidence about the similarity or differences in priorities raised by AHP, BWS, or ranking cards.

This study accompanied a research project designed to gather patient needs concerning the establishment of a central information portal about rare diseases (Zentrales Informationsportal über seltene Erkrankungen, ZIPSE). Since the available space on the website was limited, the most important information categories for patients occupy the most space followed by the less important information categories. Various information requirements on diagnosis, therapy, self-help, research, and specialized care facilities for people living with rare diseases, their relatives, and health care professionals were identified in qualitative interviews (see [19]). However, the ranking of the information criteria remained unclear. AHP was a suitable method for prioritizing these information categories in the next step (see [20]). Since AHP is a relatively new approach in health care and it is rarely been used in health care research compared to BWS and DCE, several methodological aspects remain unstandardized. Forman et al. (1998) described different aggregation methods for group decisions with the AHP method: aggregating individual judgments (AIJ) and aggregating individual priorities (AIP) by arithmetic mean or geometric mean [21]. The choice of aggregation method depends on the circumstances and the aim of the study. We wanted to examine and compare the resulting differences in decisions of the aggregation methods in our study. This paper shows outcomes for the different Consistency Ratio (CR) thresholds, aggregations methods, and sensitivity analyses. Furthermore, the study tries to identify how to validate the AHP outcomes. Outcomes were compared with the results of questionnaires using the following well established methods: BWS Case 1, and ranking cards. The first aim of this study was to demonstrate the impact of the non-standardized AHP method on priorities. Does the aggregation method influence the resulting group priority rankings? The second aim was to compare the AHP outcomes with the outcomes achieved by BWS and ranking methods to validate the resulting priorities from patient perspective (convergence validity).

## Methods

### AHP method and application

The AHP method originates from the marketing sector, invented by Thomas Saaty in the late 1970s. Dolan et al. applied the method of AHP the first time in the health care sector several years later in 1989 [22, 23]. Nevertheless, the AHP remains a rarely used decision making method in health care research compared to BWS, ranking cards, and DCE. The following methodological explanations are in accordance with Saaty [24]. The AHP decomposes the decision problem at different levels of hierarchy. The first level describes the aim of the decision making. This is then explained in further detail at a lower level using sub-criteria. The last level contains possible alternatives with their characteristics. In the interview, the participant compares all criteria pairwise at each level (15 comparisons in total) using a scale ranging from 9 to 1 to 9. Thereafter, the judgments of the pairwise comparisons set up a matrix. This method presumes that the reciprocal request results in reciprocal weights of judgments; therefore, only the upper half of the matrix has to be queried. The matrices are used to calculate weights by the Eigenvector Method. Additionally, the Consistency Ratio (CR) can be computed from the matrices to examine whether the participants’ answers are random. Following Saaty, the CR has to be ≤ 0.1. Other authors suggested a CR ≤ 0.2, but the threshold value is not defined consistently [8, 25]. Higher CR values indicate exclusion of answers and questionnaires due to inconsistency.

First, we briefly report the results of information requirements of patients with rare diseases. Second, we compare the results of CR ≤ 0.1 and CR ≤ 0.2 for median, quartiles, and extreme values (as box-plots). Third, different aggregation methods (geometric mean, arithmetic mean, and median) are used and the differences in results noted. Saaty suggested to calculate group priorities by aggregating judgments or final outcomes by geometric mean to satisfy the reciprocal property of the AHP [26]. Reciprocal properties present the first axiom for the AHP, meaning that the strength of one criterion’s dominance over a second criterion is inversely proportional to the second criterion’s dominance over the first. This implies that if criterion A is five times more important than criterion B, criterion B is one-fifth the importance of criterion A (for all axioms see [27]). This relationship must be preserved after aggregation and can be achieved by the geometric mean method. The geometric mean is always smaller than the arithmetic mean, except for one observation is zero [28]. In this sub-section, we also examine differences in the results for aggregating individual judgments (AIJ) in contrast to aggregating individual priorities (AIP). Additionally, a sensitivity analysis estimates the stability of weights. As most AHPs combine specific criteria combinations into overall alternatives (e.g., criteria combinations to describe three different cars), the sensitivity analyses focus on the stability of these alternatives. Because no standard method for the AHP without combining the attributes to alternatives was implemented, we looked at the confidence intervals (CIs) for each global weight of the criteria, and identified the stability of the ranking positions for each criterion. Therefore, we determined the BC_a_ bootstrap 95%-CI because our sample was small and in this case bootstrap CI were more accurate and correct than the standard CI [29]. All our analyses were conducted with the R statistic software program and the package “pmr” [30].

### Methodological background of the BWS and ranking cards

As a second method in this paper, we applied BWS Case 1 in the same study population population [31]. Here, different combinations of the criteria built up the sets. The interviewee selected the best and the worst criteria in each set, resulting in two decisions per set. Each person answered seven sets. The BWS method is based on random utility theory, and uses the choice models or the count analysis. Methods used in choice approaches are multinomial logit model, conditional logit model, maximum-likelihood, or weighted least square method population [31]. Since we were not interested in predictors for the decision, but rather in rankings, we emphasized the count analysis method and rankings.

Using ranking cards resulted in an ordinal ranking of criteria, implying that distances between criteria could not be measured. Besides, it was a well-established warm-up task [32], and could support the interviewee to remain consistent with their prior ranking throughout all tasks. This survey included the ranking cards method before the AHP tasks.

### Comparison of results from AHP, BWS, and ranking cards

Furthermore, the results from AHP, BWS, and ranking cards were compared. We placed the results in a table and examined differences in the rank. The AHP’s weights could not be compared with the weights from the BWS, because they are based on deviating mathematical calculation methods and scales. In addition, we conducted tests for correlation between the ranks with the help of Kendall’s tau b coefficient. This coefficient was used for rank ordered data, and identifies concordant and discordant rankings between two or more variables [33]. The Kendall’s tau b makes adjustments for ties in the data, in contrast to Kendall’s tau a.

### Survey design

The study sample consisted of randomly selected participants from the qualitative main study of the ZIPSE project [19]. A positive vote was obtained from the ethics committee of Albert-Ludwigs-University Freiburg (number 53/14). As it was an accompanying research project, inclusion and exclusion criteria for participants were equal to those of the main study sample. Therefore, participants were at least 18 years old and were either suffering from a rare disease, or were the near relative of a sick individual. In this study participants were interviewed either face-to-face, or via phone with a paper-pencil questionnaire that contained AHP, BWS, and ranking tasks. Criteria development is described in detail by Babac et al. [20]. Additionally, socio-demographic and disease specific data were collected. A ranking task of cards with the criteria’s descriptions should support consistent answering. Therefore, participants arranged the cards according to their preferred order, and left them next to the questionnaire during the rest of the interview. The interviewer indicated inconsistencies between ranking cards. Hence, participants could adjust either the order of the cards, or the judgment in the questionnaire.

Financial support for this study was provided in part by a grant from the Federal Ministry of Health. The funding agreement ensured the authors’ independence in designing the study, interpreting the data, writing, and publishing the report.

## Results

Initially, we report the AHP results including the criteria description and their hierarchical arrangement. Then, we show the information criteria priorities evaluated by patients with rare diseases or their relatives. The following subsections investigate the outcomes of different methodological approaches in the AHP method. Finally, we report the comparison of AHP results with BWS and ranking tasks.

**Fig. 1:**
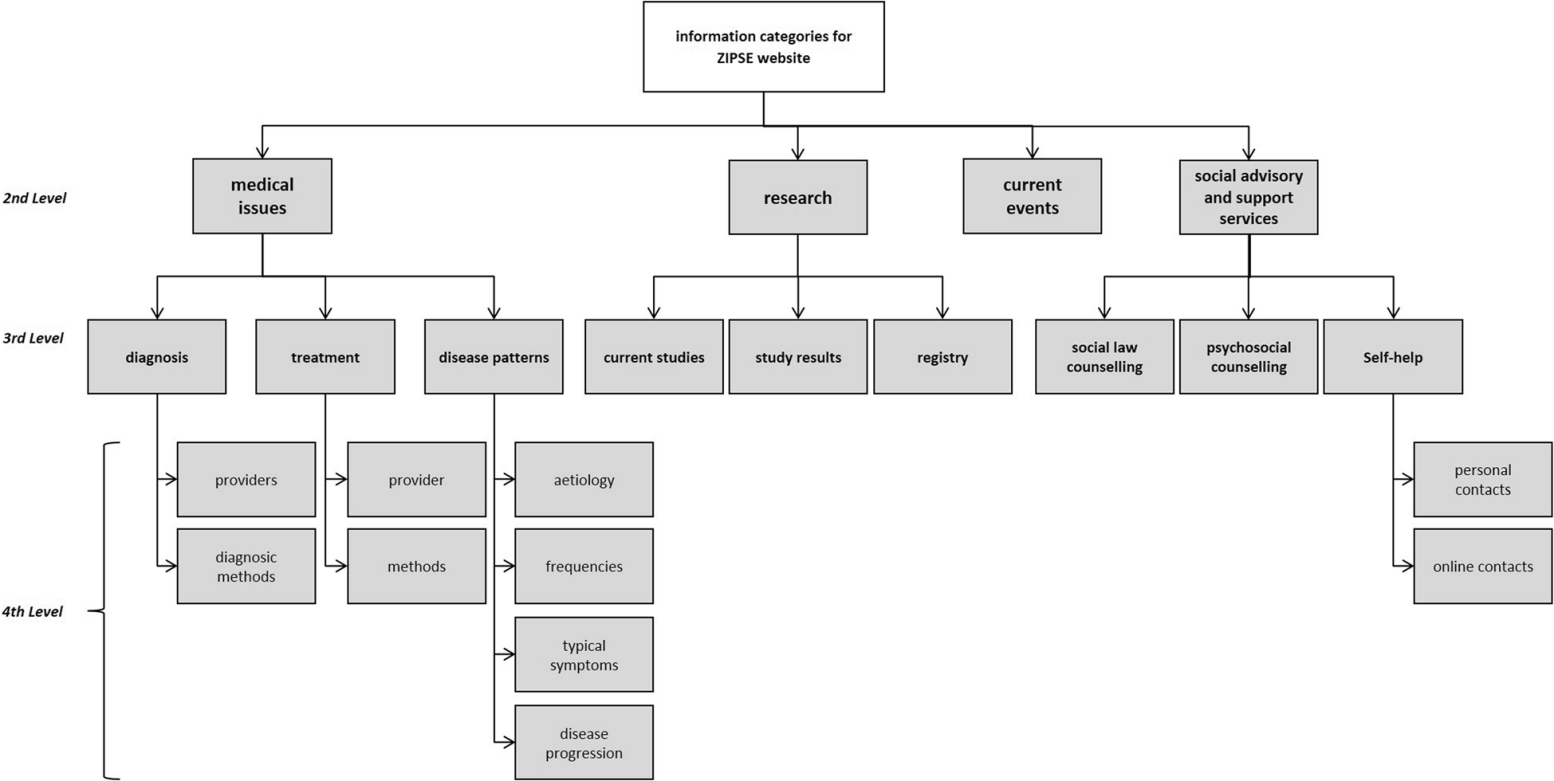
Hierarchy of rare diseases information categories

Figure 1 shows the final hierarchy for the AHP. It consists of four levels with the aim of study on the first level. The aim decomposes into information about *medical issues*, *research*, *current events,* and *social advisory and support services*. The topic of *medical issues* was again subdivided into *diagnosis*, *treatment,* and *disease patterns*. The first two were split into *provider* and *methods* at the fourth level. *Disease patterns* contained *aetiology*, *frequency*, *typical symptoms,* and *progression* at the lowest level. At the third level *research* implied *current studies*, *study results,* and *registries. Current events* at level two contained no further subcategories. The last category at level two was divided into *social law counseling*, *psychosocial counseling,* and *self-help* at level three. *Self-help* further held the subcategories of *personal contacts* and *online contacts* (fourth level). Further details and descriptions can be found in Additional file 1.

The sample for our decision analysis consisted of 31 women and 8 men with mean age of 53.82 years. The inequitable distribution of gender was due to the fact of unequal proportions in the qualitative main study.

**Fig. 2:**
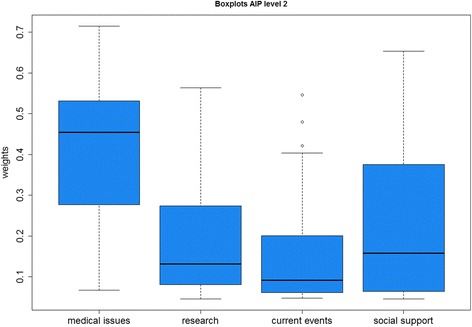
Boxplots of global weights from criteria at second level

In the first scenario, all participants who reached a CR at second level exceeding 0.1 were excluded from the analyses. Then 22 included participants (19 women, 3 men; mean age: 52.50 years) remained for further analytical steps. In this scenario, we calculated weights for each included participant and then aggregated the weights (AIP method). The first approach was aggregating the weights by median. In Fig. 2, the results are shown as boxplots including the quartiles and distribution of weights for each criterion at second level.

The boxplots show that *medical issues* were the most important criteria for the participants with a median weight of 0.4548 (SD = 0.1728), followed by *social support* (weight (w) = 0.1575, SD = 0.1777), and *research* (w = 0.1314, SD = 0.1462). The least criterion was information about *current events* with a median weight of 0.0913 (SD = 0.1550). The SDs of *social support, research,* and *current events* indicated high variations of the priorities in the sample.

**Fig. 3:**
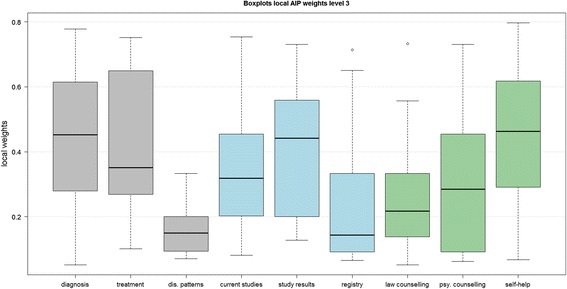
Boxplots local AIP weights at third level

Figure 3 shows the local weights of sub-criteria at the lower third level. The gray boxplots indicated the sub-criteria of *medical issues* with the highest weight for *diagnosis* (median weight (mw) = 0.4517, SD = 0.2240), followed by *treatment* (mw = 0.3512, SD = 0.2223), and *disease patterns* (mw = 0.1492, SD = 0.0763). The second information criterion of *research* (blue boxplots) included *current studies*, *study results,* and *registry*. The most important sub-criterion was *study results* with a local weight of 0.4416 (SD = 0.2015), the second *current studies* (w = 0.3184, SD = 0.1955), and the third was the information about *registries* (w = 0.1429, SD = 0.2142). The green boxplots displayed the local weights for the category of *social support. Self-help* (w = 0.4663, SD = 0.2307) reached the highest weight followed by *psychosocial counseling* (w = 0.2845, SD = 0.1801), and *law counseling* with the lowest weight of 0.2167 (SD = 0.1768). We did not compare the global weights of sub-criteria against each other because high weights at the second level (e.g., for *medical issues*) would highly influence the weights at the third level. Therefore, we used the sub-criteria’s local weights for comparisons within each criterion because the global weights were not important for our methodological considerations.

### Comparison of consistency thresholds

**Fig. 4:**
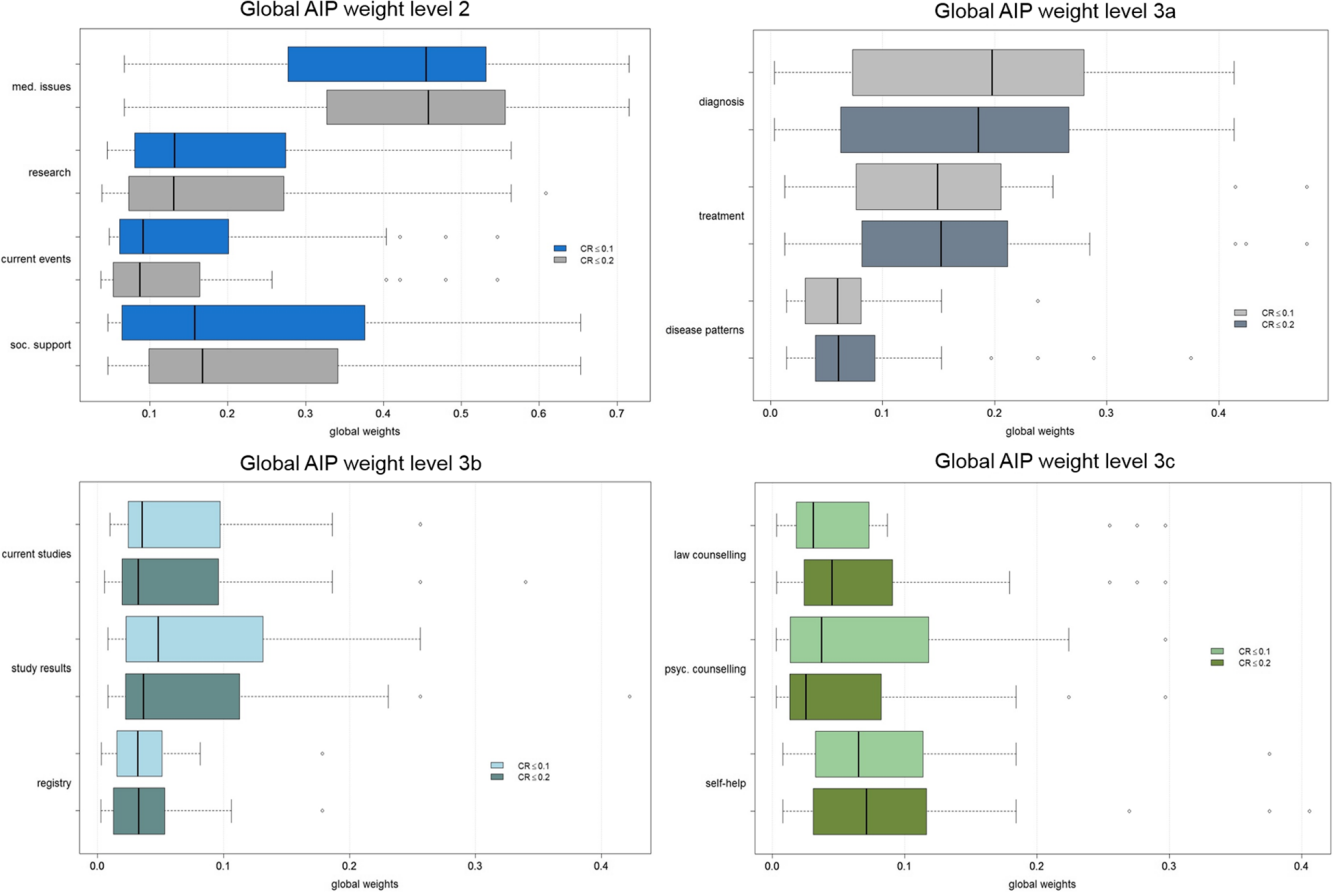
Boxplots global AIP weights separated by CR

Figure 4 shows the boxplots for all global weights separated by level. Additionally, it compares the boxplots for a threshold of included participants with high consistency (CR ≤ 0.1) and a threshold of lesser consistency (CR ≤ 0.2). All graphs show an almost equal median for the two groups of CR and a *t*-test indicate no significant differences of median for each criterion (not shown here). However, a difference in the ranking by median occurs at level three: *law counseling* gained a higher weight for an extended threshold and received rank 9 (w = 0.0310) instead of the 13th and last rank (w = 0.0452). At the same time, *psychosocial counseling* fell from rank 10 to 13 (weight 0.0372 onto 0.0254). A rank reversal occurs for *current studies* (weight 0.0353 onto 0.0324) and *registries* (weight 0.0319 onto 0.0325). In summary, the medians between a lower and a higher CR threshold did not differ significantly. Nevertheless, when small differences in weights occurred, rank reversals could be observed. In this study, rank reversals occurred only for the last four rankings.

### Comparison of aggregation methods

In the next step, we analyzed differences in global weights by different aggregation methods. All mean calculations were based on geometric mean calculation as it serves the Pareto Principle and therefore seems to be the correct approach in theory [10, 34]. In the first scenario, the AIJ was applied. This method aggregated the comparison matrices first. In a second step, priority weights were calculated for each criterion. An overall CR was calculated for level two after the aggregation of all individual opinions. In the second scenario the AIP method was applied. This methodology calculated eigenvectors and priorities for each participant first. Only participants with a CR smaller than or equal to 0.1 were included in the aggregation. Afterwards, resulting priority weights were aggregated through geometric mean calculation.

**Fig. 5:**
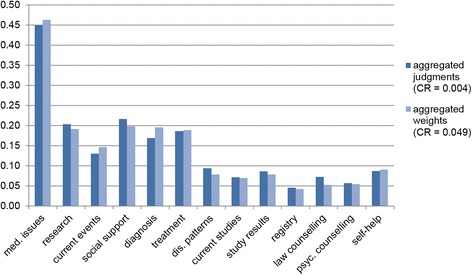
Comparison of global weights for different aggregation levels

Figure 5 displays the results of the two scenarios that comprised all 31 participants for scenario 1 and 22 for scenario 2. The aggregated judgments (scenario 1) show similar global weights for most of the criteria compared to the aggregated weights (scenario 2). Rank reversal occurrs between *diagnosis, treatment,* and *research,* because for scenario 1, *research* (w1 = 0.2038) and *treatment* (w1 = 0.1862) were more important than *diagnosis* (w1 = 0.1691), whereas in scenario 2, *research* (w2 = 0.1916) and *treatment* (w2 = 0.1892) were less important than *diagnosis* (w2 = 0.1955). Likewise, the ranking differs for *self-help, study results,* and *disease patterns*: in scenario 1, *disease patterns* (w1 = 0.0940) were more important than *self-help* (w1 = 0.0871) and *study results* (w1 = 0.0860), and in scenario 2, it was the other way round (*self-help* w2 = 0.0906, *study results* w2 = 0.0786, *disease patterns* w2 = 0.0785). A third rank reversal can be seen for the two scenarios between *current studies* (w1 = 0.0721, w2 = 0.0704, rank 11 vs. 10), *psychosocial counseling* (w1 = 0.0568, w2 = 0.0547, rank 12 vs. 11), and *law counseling* (w1 = 0.0729, w2 = 0.0531, rank 10 vs. 12). The CR for the second level was 0.004 in the first scenario, whereas the CR was 0.05 in the second scenario.

In the next step, the AIJ and AIP were compared by median. The table for these comparisons can be found in Additional file 2. The results are nearly identical to Fig. 5. The differences are small deviations in the weights and a few higher weights for the AIP than the AIJ (*current events*, *registries,* and *self-help*). The last comparison of AIP and AIJ was conducted by their means. Here, the AIP were markedly higher than most of the AIJ, also in comparison with the AIPs of the previously mentioned aggregation methods. Additionally, the weights summed up to 1 at first level, and they yielded the appropriate weights at lower levels. However, the most important question in this context was whether the ranking position changed through the different aggregation methods. Table 1 answers this question.

**Table 1:** Comparison of aggregation methods and weights

	Geometric mean ranking		Median ranking		Mean ranking	
	AIJ	AIP	AIJ	AIP	AIJ	AIP
Med. issues	1	1	1	1	1	1
Research	3	3	5	5	3	3
Current events	6	6	9	6	6	5
Social support	**2**	**2**	4	3	**7**	**2**
Diagnosis	5	4	2	2	2	4
Treatment	4	5	3	4	4	6
Disease patterns	7	8	**6**	8	9	**11**
Current studies	**11**	10	7	**11**	**5**	10
Study results	9	9	8	9	8	8
Registry	13	13	13	12	11	13
Law counseling	10	12	10	13	10	12
Psychosocial counseling	12	11	11	10	12	9
Self-help	8	**7**	12	**7**	**13**	7

The bold data highlights the results in the following text passage

The noticeable difference occurs for the criterion *self-help*, which took the ranking positions from 7 to 13 over the different methods. Another striking criterion is *current studies*, which obtains ranking positions between 5 and 11. Two less intensive varying criteria were *social support* and *disease pattern* that differed between 5 positions. The further 9 criteria varied between 3 ranking positions, so a relatively stable valuation could be assumed.

Finally, the influence of aggregation method on CR had to be examined. The CR in the scenario of aggregation by geometric mean was markedly lower for AIJ than for AIP (CR AIJ: 0.0045; CR AIP: 0.0490), although only participants with a CR ≤ 0.1 were included for the AIP. By using the median (CR AIJ: 0.0683; CR AIP: 0.0674) or mean scenario (CR AIJ: 0.0745; CR AIP: 0.0587), the CRs were similar, but still much higher than the CR from AIJ by geometric mean, as expected.

### Sensitivity analysis of AHP results

**Fig. 6:**
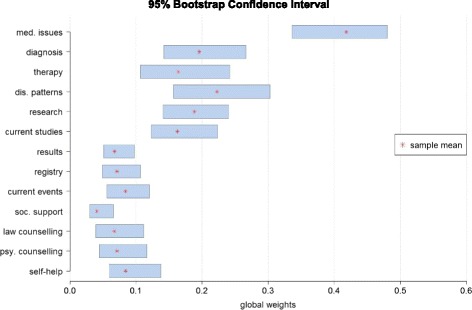
95% bootstrap confidence intervals for global weights

Usually AHP examine a combination of (sub-)criteria weights resulting in decision alternatives. Thereafter, the sensitivity of alternatives can be analyzed. However, the underlying study does not integrate a hierarchy level with decision outcomes, but only criteria and sub-criteria. Therefore, we looked at the stability of the criteria’s ranking positions. Consequently, we calculated the CIs for each global weight (see Fig. 6). In addition, we show the mean weight of the underlying sample. The CIs distributed over three ranges for global weights. The seven lowest criteria in the figure from *self-help* to *results* showed CIs from approximately 0.03 to 0.14, and the CIs were rather small, particularly *social support*. Then, the criteria of *current studies*, *research*, *disease patterns*, *therapy,* and *diagnosis* covered a CI from approximately 0.11 to 0.30. A markedly higher CI arose for *medical issues* (CI: 0.34–0.49). It could be concluded that within the first two groups, the criteria were likely vulnerable to rank reversal. In contrast, the first rank for *medical issues* was assumed to be robust.

### Comparison of methods

In the next section, we wanted to contrast the results of the AHP and the BWS. Table 2 compares the results of the methods. The most important criterion at level two was information about *medical issues* in all three methods, followed by *social support* and *research*. The least important criterion, *current events*, was also equal for AHP and BWS, but for the ranking cards it was also ranked position 3. At level three for *medical issues*, the most important criterion was *treatment* in the BWS, and diagnosis in the AHP. *Disease patterns* took the third position in both cases. The sub-criteria for *research* were ranked as followed for BWS and also AHP: 1) *study results*, 2) *current studies*, 3) *registry*. In the category of *social support,* the most important sub-criterion was *self-help*. The positions 2 and 3 differed between BWS and AHP. In the BWS, the second important sub-criterion was *law counseling,* whereas it was *psychosocial counseling* in the AHP. The ranking cards results showed doubled ranking positions at all levels, particularly when BWS and AHP were indifferent.

**Table 2:** Comparison of BWS, AHP, and ranking cards

Criteria	BWS values	AHP local weights	BWS ranking	AHP ranking	Ranking cards^a^
Med. issues	1.000	0.368	1	1	1
Research	0.322	0.152	3	3	3
Current events	0.000	0.117	4	4	3
Social support	0.372	0.158	2	2	2
Diagnosis	0.855	0.354	2	1	1
Treatment	1.000	0.342	1	2	1
Dis. patterns	0.000	0.142	3	3	2
Current studies	0.279	0.304	2	2	2
Study results	1.000	0.339	1	1	1
Registry	0.000	0.184	3	3	2
Law counseling	0.421	0.213	2	3	2
Psyc. counseling	0.000	0.220	3	2	2
Self-help	1.000	0.363	1	1	1

^a^Equal ranking for multiple criteria permitted

Because the ranking cards gave orientation for the AHP in the interviews, we assumed that there was a correlation between their results. Therefore, we did not evaluate the correlations for AHP and ranking. We examined the correlation between AHP and BWS rankings by Kendall’s tau coefficient, for each hierarchical level. We found significant moderate to strong correlation between the two methods in the rankings (see Table 3).

**Table 3:** Correlation between AHP ranking and BWS ranking for each level

	Kendalls tau	*p*-value
Level two	0.585	<0.001
Level three a	0.543	<0.001
Level three b	0.613	<0.001
Level three c	0.668	<0.001

## Discussion

In this paper, we focused on methodological aspects of AHP and comparison of methods. The first step was to compare the results for different CR thresholds. Thereby, we considered the weights for including all interviewees with CR ≤ 0.1 or CR ≤ 0.2. We found that the mean weights between these two groups did not differ significantly. However, rank reversal could occur if the criteria’s weights are close. For clarification, another phenomenon in AHP is also called “rank reversal”: it occurs when adding or deleting an alternative leads to a shift in the previous alternatives’ ranking order [35, 36]. The latter phenomenon was not investigated in our study.

The second step was to compare different aggregation methods. Therefore, we calculated the geometric means of the AIJ method (scenario 1) as well as the AIP method (scenario 2). The first difference was the number of participants that were included with a CR ≤ 0.1. In the first scenario, we included 31 participants, and in the second scenario, we had to exclude 9 participants because they showed CRs > 0.1. In the first scenario, we had a CR of 0.004 for the second level calculated after aggregating the judgments. In the second scenario, the CR at the second level was 0.05, and thus higher than in scenario 1, although the participants with CRs > 0.1 were excluded from the final CR calculation. The results received from scenario 1 showed almost the same weights compared to the results from scenario 2. Besides, the criteria’s rankings differed between the scenarios, due to short distances between the weights. The AIJ method implies that the group decides as a new individual whereas the AIP method is based on the assumption that each individual decides on her or his own and the resulting decisions are aggregated [21]. Therefore, the aggregating method should depend on whether the sample is seen as one unit or a group of individuals. Forman et al. (1998) argued that for AIJ the geometric mean must be used because otherwise two social choice theory axioms (Pareto optimality and homogeneity) are not satisfied [21, 37]. The Pareto optimality axiom describes that the most frequently preferred alternative in the individual decisions must be the preferred one in the group decision. The homogeneity axiom states that the ratio between the criteria weights is the same for individual and aggregated group judgments. Our study supported Forman’s demand as we saw violations of the Pareto axiom in Table 1, but not for the most preferred criterion. The homogeneity axiom was not investigated in our study. In future AHP studies, following Forman et al. (1998) and Saaty (2008) the geometric mean should be used in AIJ method.

In the third step, we opposed the criteria’s rankings received from aggregated weights and judgments by geometric mean, median, and mean. Here, the ranking positions showed deviating results and rank reversals. These aspects should be considered when results derived by different aggregation methods in studies are compared.

As no sensitivity analysis is suggested for AHPs that do not include alternatives, we tried to find an appropriate one. The aim of sensitivity analysis in AHP is to find instable criteria that could cause rank reversal. Therefore, we illustrated the 95%-CIs for all criteria. Where CIs overlap because of similar weights, the risk for rank reversal increased.

Finally, we evaluated the criteria’s rankings for the different methods (AHP, BWS, ranking cards). However, we could not compare the weights from AHP with the weights from the BWS, because they use different scales. Therefore, only the rankings could be compared between the methods. Here, we found moderate to strong correlations between the AHP and BWS.

Correlated results between the methods were similarly reported by prior studies. Pignone et al. (2012) investigated differences in value elicitations with CA, rating, and ranking tasks [38]. They concluded that the CA produced different values compared with ranking and rating, but the latter two led to similar results. Van Til et al. analyzed the differences between pairwise comparisons, BWS, five point rating scales, point allocation and ranking [39]. There were no differences between the methods at group level; however, differences occurred at the individual level and the largest differences were between pairwise comparisons and the five point rating scale. The correlation between the methods for individual weights was moderate. Furthermore, the order of the methods shown in the questionnaire influenced the weights. We did not examine this aspect in our study, because we had a small sample, and could not expect significant results regarding this question. Therefore, the order of tasks could also influence the results.

A major problem was the inconsistent response behavior of the participants in the AHP. Our sample consisted of patients with different rare diseases. The diverse clinical pictures and disease stages could have led to different priorities in the evaluation of the information criteria. Although in our study the participants used ranking cards for assistance during the AHP, the CRs were not all below the defined threshold. This phenomenon raised the question, whether the AHP method was not applicable in certain participant groups or in a heterogeneous sample. Therefore, future research projects should investigate the requirements for their participants, because this could bias the results. Further studies should also examine whether the aggregation of judgments always leads to higher values than the aggregation of weights, as detected in our study.

Another aspect was the small number of participants. Although we neglected this aspect in our study, the number of participants could also be an influencing factor of the results. Recent literature suggests that AHP is particularly useful for small groups, because priorities can be calculated for each participant [40]. As we used the sample from the main study, a larger proportion of women was included. Nevertheless, by aggregating the individual judgments or weights the researcher gave a statement for a (heterogeneous) group. Thus, we should present the results from the AHP under the restriction of their study population. The results were representative for this study population only.

## Conclusion

In the AHP method, the number of patients is influenced by the CR aggregation method and the threshold of the CR, which could bias the results. Therefore, it is important to establish guidelines and investigate the differences for each study as also mentioned by Schmidt (2015) [7]. The comparison between the different methods (AHP, BWS, ranking tasks) resulted in similar outcomes.

The AHP seemed to be a challenge for some participants. Reasons could be the unusual scale and the need for consistency over several questions. However, we could not identify special groups because our sample was too small and homogenous. The BWS also forced the participants to make decisions. However, here only the best and worst decision had to be made. Therefore, the cognitive burden is reduced compared to other methods, for example, the DCE [41]. The researcher should consider the trade-off between methods that are easy to understand, and the method’s gain of information as well as the method’s theoretical basis. In addition, the sensitivity of each method should be calculated for each research question. In sum, the choice of method depends on the trade-off between the burden for participants and possibilities for analyses. Consequently, the method should be chosen according to the characteristics of the study sample and the aim of the study.

## Additional files

Additional file 1: Description of the AHP criteria. (DOCX 15 kb)
Additional file 2: Aggregation level and different means. (DOCX 13 kb)

## Abbreviations

AHP: Analytic Hierarchy Process
AIJ: Aggregation by individual judgment
AIP: Aggregation by individual priority
BWS: Best-worst-scaling
CA: Conjoint analyses
CI: Confidence interval
CR: Consistency ratio
DCE: Discrete choice experiment
IQWiG: Institute for Quality and Efficiency in Health Care
MW: Median weight
SD: Standard deviation
W: Weight
ZIPSE: Zentrales Informationsportal über seltene Erkrankungen (English: central information portal about rare diseases).
