# Supplementary material for: Measuring patients’ priorities using the Analytic Hierarchy Process in comparison with Best-Worst-Scaling and rating cards: methodological aspects and ranking tasks
Source: Health Econ Rev. 2016 Nov 14;6:50. doi: 10.1186/s13561-016-0130-6 (PMC5108732; doi:10.1186/s13561-016-0130-6)
Supplement: Additional file 1: — Description of the AHP criteria. (DOCX 15 kb) [file 13561_2016_130_MOESM1_ESM.docx]

Appendix 1: Description of the AHP criteria

| 1. **Medical Issues**   Information that explains the medical background of a rare disease. These include, for example, information about diagnosis, therapy, or development of the rare disease.   - 1. **Diagnosis:**      - *Health care providers:* Names, addresses, phone numbers, specialized providers‘ websites for diagnosis of rare diseases.      - *Methods:* Used to detect rare diseases, process of diagnosis.   2. **Treatment:**      - *Health care providers:* Names, addresses, phone numbers, specialized providers‘ websites for therapy of rare diseases (e.g., physicians, therapists).      - *Therapy process:* Information that describes the process of therapy (e.g., surgery, drugs).   3. **Disease patterns:**      - *Aetiology:* Cause of the illness, if known. An example for causes of rare disease is genetic defects.      - *Frequency:* Number of persons suffering from this particular rare disease.      - *Typical symptoms:* Information about symptoms and typical signs of the disease*.*      - *Progression:* Change in symptoms and disease severity over time and difference in disease stages. |
| --- |
| 1. **Research**   The second criterion gives information on research. Here you can find news about outcomes in pharmaceutical research or scientific research results within the scope of rare diseases.   - 1. **Current studies:**   Studies in field of rare diseases that are currently searching for study participants.   - 1. **Study results:**   Latest findings from medical research.   - 1. **Registries:**   Storing long term data. Registries aim to improve therapies, distribution, and disease control. You can also find enrolment information. |
| 1. **Current events**   This topic covers joint actions for patients and health care staff. The events can be used to share experiences and information and assess public perception (e.g., date of rare disease day). |
| 1. **Social advisory and support services**   Here you can find contact data about information centers that are specialized in helping rare disease patients. They provide counseling in topics like applications and legal rights, as well as information and counseling regarding psychological stress or self-help.   - 1. **Social law counseling:**   This subcriterion deals with questions regarding health insurance services, labor law, pension scheme, and others. Here, all your questions about requests/applications and reimbursement issues are answered.   - 1. **Psychosocial counseling:**   Information and contacts about counseling services for patients with rare diseases and their relatives as well as friends and colleagues.   - 1. **Self-help:**      - *Personal contacts:* Contact details for local self-help groups.      - *Online contacts:* Information about websites, online forums, and email addresses to share experiences between people with rare diseases. You can also find experience reports from people with rare diseases or their relatives. |
