# Supplementary material for: Measuring patients’ priorities using the Analytic Hierarchy Process in comparison with Best-Worst-Scaling and rating cards: methodological aspects and ranking tasks
Source: Health Econ Rev. 2016 Nov 14;6:50. doi: 10.1186/s13561-016-0130-6 (PMC5108732; doi:10.1186/s13561-016-0130-6)
Supplement: Additional file 2: — Aggregation level and different means. (DOCX 13 kb) [file 13561_2016_130_MOESM2_ESM.docx]

Appendix 2: Aggregation level and different means

| aggr. level  criteria | **geometric mean** | | **median** | | **mean** | |
| --- | --- | --- | --- | --- | --- | --- |
|  | AIJ | AIP | AIJ | AIP | AIJ | AIP |
| med. issues | 0.4492 | 0.3676 | 0.5608 | 0.4548 | 0.5445 | 0.4181 |
| research | 0.2038 | 0.1520 | 0.1802 | 0.1314 | 0.2459 | 0.1956 |
| current events | 0.1302 | 0.1165 | 0.0685 | 0.0913 | 0.1063 | 0.1640 |
| social support | 0.2167 | 0.1575 | 0.1906 | 0.1575 | 0.1033 | 0.2223 |
| diagnosis | 0.1691 | 0.1299 | 0.2403 | 0.1976 | 0.2955 | 0.1883 |
| treatment | 0.1862 | 0.1258 | 0.2403 | 0.1489 | 0.1776 | 0.1625 |
| dis. patterns | 0.0940 | 0.0522 | 0.0801 | 0.0599 | 0.0714 | 0.0673 |
| current studies | 0.0721 | 0.0462 | 0.0772 | 0.0353 | 0.1274 | 0.0711 |
| study results | 0.0860 | 0.0516 | 0.0772 | 0.0482 | 0.0842 | 0.0839 |
| registry | 0.0458 | 0.0279 | 0.0257 | 0.0319 | 0.0344 | 0.0406 |
| law counseling | 0.0729 | 0.0336 | 0.0635 | 0.0310 | 0.0558 | 0.0670 |
| psyc. counseling | 0.0568 | 0.0346 | 0.0635 | 0.0372 | 0.0268 | 0.0712 |
| self-help | 0.0871 | 0.0572 | 0.0635 | 0.0652 | 0.0208 | 0.0842 |
| CR | 0.0045 | 0.0490 | 0.0683 | 0.0674 | 0.0745 | 0.0587 |
